# Coalition Game-Based Secure and Effective Clustering Communication in Vehicular Cyber-Physical System (VCPS)

**DOI:** 10.3390/s17030475

**Published:** 2017-02-27

**Authors:** Yan Huo, Wei Dong, Jin Qian, Tao Jing

**Affiliations:** School of Electronics and Information Engineering, Beijing Jiaotong University, Beijing 100044, China; 12111020@bjtu.edu.cn (J.Q.); tjing@bjtu.edu.cn (T.J.)

**Keywords:** VCPS, coalition formation game, Nash-stable, cluster stability, cluster efficiency, malicious nodes, incentive and penalty mechanism

## Abstract

In this paper, we address the low efficiency of cluster-based communication for the crossroad scenario in the Vehicular Cyber-Physical System (VCPS), which is due to the overload of the cluster head resulting from a large number of transmission bandwidth requirements. After formulating the issue as a coalition formation game, a coalition-based clustering strategy is proposed, which could converge into a Nash-stable partition to accomplish the clustering formation process. In the proposed strategy, the coalition utility is formulated by the relative velocity, relative position and the bandwidth availability ratio of vehicles among the cluster. Employing the coalition utility, the vehicles are denoted as the nodes that make the decision whether to switch to a new coalition or stay in the current coalition. Based on this, we can make full use of the bandwidth provided by cluster head under the requirement of clustering stability. Nevertheless, there exist selfish nodes during the clustering formation, so as to intend to benefit from networks. This behavior may degrade the communication quality and even destroy the cluster. Thus, we also present a reputation-based incentive and penalty mechanism to stop the selfish nodes from entering clusters. Numerical simulation results show that our strategy, CG-SECC, takes on a better performance for the tradeoff between the stability and efficiency of clustering communication. Besides, a case study demonstrates that the proposed incentive and penalty mechanism can play an important role in discovering and removing malicious nodes.

## 1. Introduction

A Cyber-Physical System (CPS) [1,2] is a complex embedded network system combining computing, communications and control on the basis of environmental sensing. In CPS, we exploit various sensors [3] to collect and transmit the sensing data for the purpose of achieving intelligent control. From this point of view, the sensor network, a typical application of CPS, is widely studied and used in all walks of life. Nowadays, as one of the most important technologies, the Vehicular Cyber-Physical System (VCPS) [4,5,6,7] has been proposed to take advantage of the latest advances in sensing, computing, communications and networking technologies to improve the safety, efficiency and resilience of transport systems. The system consists of moving vehicles and fixed Roadside Units (RSUs) [8], where various sensor nodes that are deployed on either vehicles or RSUs will acquire and share the traffic information.

However, the communication models, schemes and strategies in CPS cannot be directly used in VCPS, because of the following characteristics [9]. First, since the high speed of vehicles may lead to frequent and rapid changes of the network topology, the lifetime of the network is short, as well as the communication quality is degraded, even the disconnection in the network. Second, the channel quality is vulnerable to the complex and changeable environment and various factors, such as various topologies of roadside entities, time-varying road situations and the relative velocity among different vehicles. Third, the high traffic density can result in huge overhead in the network. Therefore, the typical characteristics of VCPS can bring about difficulties and challenges on information transmission and efficient protocols.

In order to address the challenges, the clustering network topology can be applied by taking advantage of the layout of the roadside. That is, the movement of vehicles in VCPS is more regular than that of nodes in other ad hoc networks due to the layout of the roadside. The main idea of clustering is to group a set of similar nodes and select one node as the cluster head for every group based on some criteria to achieve reliable and effective communication. Employing the relay communication in the intra- or inter-cluster via the cluster heads, the stable communication can be implemented for the complex and changeable VCPS network environment.

Although clustering schemes have already been studied in the past, most of them only concern the scenario of a highway or straight lanes. When it comes to crossroads in an urban scenario [10,11,12], however, the previous schemes have not been considered comprehensively and may lead to worse performance (e.g., the degradation and deterioration of communication quality). Obviously, we should exploit the special properties of crossroads in VCPS, such as the variable vehicle travel direction and the high throughput requirements of cluster heads resulting from the large number of vehicles, to handle and improve the transmission quality. Specifically, such a large number vehicles will cause the high bandwidth demand provided by cluster heads, and the changeable travel direction exerts an influence on the clusters’ stability, as well.

To deal with these problems, considering multiple metrics, including the vehicles velocity, position, direction and transmission bandwidth, an alternative clustering approach is presented in this paper based on coalitional game theory [13]. Specifically, we exploit the coalition formation game to model the clustering formation process. Then, the coalition as a cluster with high transmission efficiency [14] is formed with nodes that have similar mobility. Through this scheme, each vehicle will make its own decision on which coalitions to join. Furthermore, the proposed strategy is concerned with both the stability and efficiency [15] for clusters in crossroads and should reach a stable coalition structure. After accomplishing the formation of the clustering structure, we present two criteria to lower the computational complexity of the clustering maintenance mechanism.

Nevertheless, we note that some vehicles, also called malicious nodes [16,17], can tell a lie to benefit from the clusters. They may report a false provided bandwidth that they do not have so as to occupy more resources. Obviously, those fraud behaviors will result in serious degradation in the aspect of communication efficiency or even communication interruption. Thus, the selfishness issue prompts us to present a mechanism to stop such fraud behavior. That is, the system should record and reject the malicious nodes if their reputation is always low. In short, the main contributions of this paper are four-fold.

- Taking into account both stability and efficiency for the clustering communication, we propose a coalition game-based clustering strategy for the crossroads in VCPS.
- In order to take full advantage of the bandwidth resource, we exploit Bandwidth on Demand (BoD) to allocate bandwidth resource for nodes. Besides, we also take into account the transmission capacity for choosing the cluster heads.
- Two criteria are introduced into the proposed cluster maintenance scheme to find the potential heads for the purpose of reducing the number of iterations and the calculation.
- A reputation-based incentive and penalty mechanism has been proposed to report and record malicious nodes, so as to ensure system reliability, as well as enhance the clusters’ average throughput.

The rest of this paper is organized as follows. The related works are described in Section 2. Section 3 pinpoints the network model and depicts the metrics for the clustering strategy. Accordingly, we define the utility function of the coalition formation game, demonstrate the associated main properties of the game and present the specific coalition algorithm, including the clustering formation algorithm and the maintenance algorithm in Section 4 and Section 5. Section 6 proposes a reputation-based incentive and penalty mechanism. Sequentially, we present the numerical analysis to evaluate the penalty of our strategy in Section 7 and conclude our paper in Section 8.

## 2. Related Work

The VCPS communications have attracted great interest in the past few decades. Many strategies and schemes were developed and presented for ensuring the application and controlling data transmission in the network. Game theory, as an important mathematical method to learn behavioral relations and make logical decisions, was widely used in VCPS. The work in [18,19,20,21] studied how to stimulate message forwarding in VCPS based on coalitional game theory. It proposed an incentive scheme for VCPS taking the source vehicles into consideration. The work in [21] addressed the popular content distribution problem in a highway scenario by means of the coalition formation algorithm, in which popular files were distributed to a group of on-board units. In [22], the authors analyzed the cooperative service-based message sharing problem in VCPS. Some nodes within a coalition could work as a relay, which was modeled as a formation game to select exactly one relay among a group of potential relay nodes to improve the efficiency of the network. Based on the research before, we will apply the coalitional game theory to design the clustering strategy in VCPS for the purpose of achieving more stable and efficient data transmission.

The clustering strategy is a process of grouping nodes together according to pre-defined rules, which had been already extensively researched in the past. Here, we briefly review the works related to clustering methods first. One of the most frequently-mentioned clustering algorithm, MOBIC [23], was a clustering algorithm initially designed for CPS, which could be also applied to VCPS. It was a classical and typical method to choose a cluster head based on the mobility metric that was computed by the ratio between the successive measurements of received power at any node from its neighbors. However, the performance of MOBIC was poor for the vehicular network because of the fast and variable characteristics in the more complex scenario. To improve the performance of clustering transmission in VCPS, the mobility-based clustering algorithm was put forward. Introduced in [24], the algorithm divided the vehicles into different speed groups that helped to identify various clusters. However, the quality of information transmission via [24] was limited due to the remote transmission. Using the vehicle destination as the key factor in [25,26], the clustering algorithms attempted to accurately describe and simulate the mobility pattern of the network. There might be a problem with knowing the final destination a priori as drivers usually do not use the navigation system for known routes. Then, the work in [27] was formed only on the basis of the node with its stable neighbors by using the defined metrics of velocity, movement direction and position.

Although the algorithm in [23,24,25,26,27] was designed for the VCPS, there still exists some isolated vehicles when the velocity of one vehicle in one cluster deviates too much from the velocity of other vehicles in the same cluster, which should result in the unstable clustering network. Additionally, the aforementioned clustering schemes were usually suitable for either the highway scenarios or the straight lane scenarios to achieve the relay-based data transmission. Still, there were only a few works to discuss the clustering communication for the crossroad scenarios. Among these works, [28] presented the EnLOSCscheme to ensure the stability and security of clusters and to reduce the communication overhead. However, EnLOSC scarcely considered the transmission capacity of cluster heads and the bandwidth demands of members, which could lead to low efficiency.

Different from these works, our strategy focuses on the balance achieved between stable clusters and efficient transmission and exploits the coalition formation game to solve the issues of clustering formation and maintenance. Additionally, the malicious nodes in clusters will also heavily decrease the communication efficiency. A great number of reputation and trust mechanisms [29,30] has been suggested and studied in order to detect and eliminate selfish nodes in wireless networks. Therefore, we also study the selfishness and present an incentive and penalty mechanism to improve the security of the clusters in VCPS.

## 3. Network Model

### 3.1. System Model

**Figure 1:**
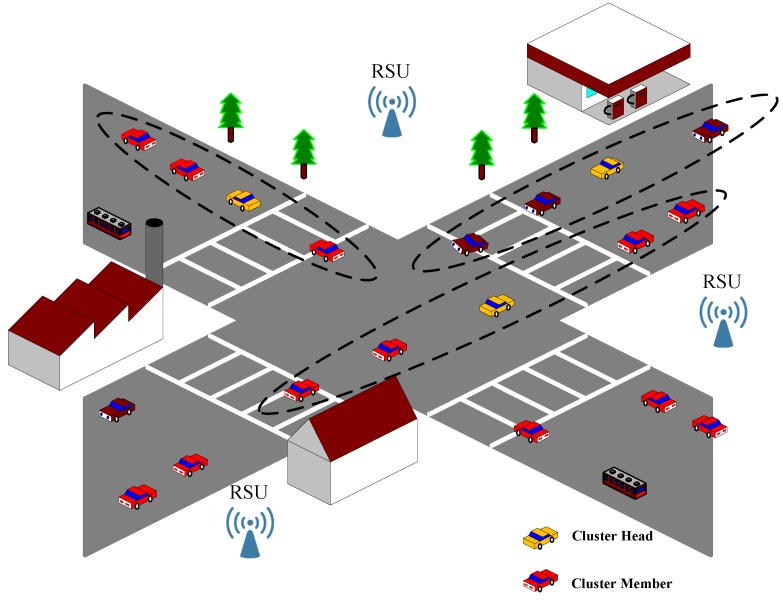
System model.

In this paper, we consider a bidirectional multi-lane city road scenario with a crossroad where various clusters have been formed from adjacent vehicles in specific regions, as illustrated in Figure 1. In every cluster, a Cluster Header (CH) is considered as the vital node to be responsible for the inter-cluster and intra-cluster communication. Basically, a CH should be stable and has a higher transmission capacity with respect to the Cluster Members (CMs). Since the process of clustering requires interaction between the vehicles, we describe the stability and the transmission capacity for each vehicle by the relative velocity, the relative position and the available bandwidth in the model. However, some vehicles may attempt to benefit their own transmission by lying about their real transmission capacity. Besides, a selfish cluster head may degrade or even destroy the whole cluster communication. Therefore, the static infrastructures, called Roadside Units (RSUs), are deployed to mitigate the adverse effects of those malicious nodes as shown in Figure 1.

### 3.2. Metrics for Clustering Strategy

We assume all vehicles are equipped with GPS so that each of them is aware of its own velocity, position and direction sensing information at any time. If a node wants to join a cluster, it has to move in the same direction with the CH. The direction vector of node *i* is expressed as $\vec{D_{i}}=D_{ix}\cdot \vec{X}+D_{iy}\cdot \vec{Y}$, where $\vec{X}$ and $\vec{Y}$ are the unit vectors of the *X* and *Y* axes. Accordingly, the angle between the directions of nodes *i* and *j* is:

(1) $$\theta_{i,j}=arccos\frac{\vec{D_{i}}\cdot \vec{D_{j}}}{\left|\vec{D_{i}}\right|\left|\vec{D_{j}}\right|}$$

In a practical environment, we consider that node *i* and node *j* are moving in the same direction when $\theta <\frac{\pi }{4}$ while moving in different directions when $\theta >\frac{\pi }{4}$.

In addition to the direction of vehicle travel, the value of the relative velocity also affects the clustering scheme. Assuming i, jare in the same cluster and $V_{i}$ and $V_{j}$ as the velocity of nodes *i* and *j* obtained from GPS, we employ the Relative Velocity Metric (RVM) to indicate the relative mobility of node *i* as below.

(2) $$RVM\left(i\right)=\frac{1}{n-1}\Sigma_{i\neq j}\left|V_{i}-V_{j}\right|$$

Similarly, let $\left(X_{i},Y_{i}\right)$ and $\left(X_{j},Y_{j}\right)$ represent the GPS position of nodes *i* and *j*. The Relative Position Metric (RPM) of node *i* is calculated as follows, which is associated with the transmission path loss, as well as the lifetime of a cluster.

(3) $$RPM\left(i\right)=\frac{1}{n-1}\Sigma_{i\neq j}({\left(X_{i}-X_{j}\right)}^{2}+{{\left(Y_{i}-Y_{j}\right)}^{2})}^{\frac{1}{2}}$$

We can conclude that a node with a small RVM is likely to stay in its current cluster for a long time, while a node with a smaller RPM can have a better transmission quality with nodes in its cluster because of the short communication distance.

As proposed in [28], we define a stability metric to describe the stability of a node in one cluster:

(4) $$M\left(i\right)=k_{1}*RVM\left(i\right)+k_{2}*RPM\left(i\right)$$

According to the above analysis, we learn that a smaller M(i) in (4) indicates that *i* is more stable with its cluster nodes. Clearly, there exists a threshold $M_{0}$ to identify whether a node can be considered as a cluster head. Specifically, if M(i) of one node is smaller than $M_{0}$, we believe that it is stable enough to be a cluster head. Otherwise, the node can only be treated as a cluster member. The specific use of (4) is described in the coalition utility function in Section 4 and the head election algorithm in Section 5.

Intuitively, the huge amount of data gathered by various sensors and forwarded in the VCPS has the potential to address intersection collisions, as well as lane changing issues. Furthermore, based on the real-time sensing of ambient states by the sensors installed in the vehicles and roadside units, many information service applications can be carried out in VCPS, such as advertising, traffic control or emergency road service. Due to the increasing demand for the bandwidth of those applications, especially in a great deal of vehicles in crossroads, the real bandwidth provided by the CH may not satisfy the demand within the cluster if we use the algorithm proposed in [13,28]. In the case, we intend to allocate the bandwidth resources based on the principle of bandwidth on demand. In other words, the cluster head will allocate bandwidth for every cluster member according to its demand, which indicates that the member could finish its data transmission via the lowest bandwidth. In the following section, we exploit $B_{0}$ to describe the available bandwidth provided by the cluster head and $b_{i}$ to represent the bandwidth demand of node *i*.

## 4. Coalition Formation Game

For the purpose of achieving stable and secure communication in a crossroad, we present the analysis of the aforementioned problem via a coalition formation game. In the game, we define that vehicles in the system model are players making distributed decisions on joining a coalition. In fact, the decisions affect the social utility representing the sum of all coalition utility and introduced in Section 5. Here, we first describe the coalition utility that is related with three sub-functions, including the velocity function, position function and efficiency function, and then prove that our model satisfies the four properties of the coalition formation game.

### 4.1. Utility Function

As we know, the coalition with better utility indicates that the nodes in it have similar mobility and a better resource utility rate. Assuming that there exists a coalition with a head *h* and n-1 members, we use the velocity, position and efficiency function to represent the coalition utility. The three important sub-functions are explained as follows.

#### 4.1.1. Stability-Related Functions

The stability concept of a cluster is defined by the distance and relative velocity among the nodes in the same cluster. In other words, a more stable cluster can be formed if the nodes’ velocity and position are more similar. To obtain the stability of a cluster, therefore, the definitions of the velocity function and position function are as below.

**Definition** **1.** *Velocity function: In VCPS, the velocity function demonstrates the average relative velocity of any two moving vehicles in the same cluster. The vehicles with similar velocity are more likely to cluster together, which means that the cluster with the smaller average relative velocity is more stable. Accordingly, the velocity function is calculated by*:

(5) $$V_{hcluster}=\frac{\sum_{i=1}^{n}RVM\left(i\right)}{n}$$

**Definition** **2.** *Position function: The average distance is related to the channel quality and path loss between two vehicles. Specifically, a coalition with a smaller position function value can have a shorter average distance and better transmission quality. Similarly, the position function is computed by*:

(6) $$P_{hcluster}=\frac{\sum_{i=1}^{n}PVM\left(i\right)}{n}$$

#### 4.1.2. Efficiency-Related Function

Due to the numerous applications ranging from car safety, transportation efficiency to car entertainment and information interaction, the bandwidth demand of vehicles is various. However, the scarce bandwidth resource introduced in the previous section will result in network congestion and the degradation of communication quality. In order to take full advantage of bandwidth resource, another sub-function with regard to efficiency is defined as follows.

**Definition** **3.** *Efficiency function: Assume that we exploit the Bandwidth on Demand (BoD) that can decrease the occupation of the bandwidth to improve the utilization ratio of network resources. The efficiency function is obtained by the ratio between the bandwidth provided by the cluster head and the total demand bandwidth of cluster members, which is shown as follows*.

(7) $$E_{hcluster}=\frac{B_{0}}{\sum_{i\in hcluster}b_{i}}\left(when,B_{0}⩾\sum_{i\in hcluster}b_{i}\right)$$

To ensure that nodes in a coalition could transmit information successfully, the provided bandwidth must satisfy the requirements of all of the cluster members, which has to satisfy two criteria. First, it is meaningless when $E_{hcluster}$ is less than one, while second, $E_{hcluster}$ should be as small as possible to improve the utilization ratio of bandwidth.

Taking both stability and efficiency into consideration, the utility of coalition *h* belongs to can be defined as shown in (8), where *a* is an adjustment factor whose value should be associated with the crossroads environment. Actually, it is important to select the appropriate values of $k_{1}$, $k_{2}$ and *a* to achieve the excellent clustering performance, i.e., the optimal utility, such as using the learning automata in [31] and genetic algorithm in [32]. To simplify the calculation, here we continue to exploit the classification method in our previous work [33].

(8) $$U_{hcluster}=k_{1}*V_{hcluster}+k_{2}*P_{hcluster}+a*E_{hcluster}$$

Similar to [33,34], $k_{1}$ and $k_{2}$ are set to one and 0.1, respectively. Yet, a different *a* represents different transmission efficiency classification for the clustering scenario. Therefore, we need to determine the appropriate *a* to satisfy the changes of various scenarios dynamically to obtain an outstanding clustering structure. Comparing with [33], here, we intend to enhance the clustering efficiency as much as possible in the case of the stable clustering communication. Specifically, RSUs will exploit the regression method in (9) to adaptively adjust *a* for preferable clustering performance.

(9) $$a_{i+1}=a_{i}+\frac{E_{i}}{E_{i-1}}\delta$$

where $E_{i}$ is the *i*-th clustering efficiency and $\frac{E_{i}}{E_{i-1}}$ denotes the adjusting scale to accelerate the convergence of *a*’s calculation. Besides, *δ* represents the unit step for increasing (or decreasing) adjustment. In detail,

(10) $$\delta ≜\left\{\begin{array}{cc} \delta_{0}, & “S_{i}>S_{\theta }”\;\mathrm{and}\;“E_{i}<E_{i-1}<E_{i-2}\;\mathrm{or}\;a_{i}>a_{i-1}”\\ -\delta_{0}, & “S_{i}<S_{\theta }”\\ 0, & otherwise \end{array}\right.$$

where $\delta_{0}$ is the predefined adjustment step and $S_{\theta }$ represents the clustering stability defined in [33]. In (10), we consider that *δ* should be set to a negative value if the clustering stability is lower than $S_{\theta }$. When meeting the requirement of $S_{\theta }$, however, *δ* starts to be set to the positive $\delta_{0}$ if the clustering efficiency has declined for two continuous cycles.

After determining the value of *a*, we can calculate the specific coalition utility for every coalition (i.e., every cluster). From (8), we learn that a coalition with smaller utility is more stable and has a higher bandwidth utilization ratio. Particularly, a coalition cannot be formed if it is stable, but not efficient, and vice versa. For every node, which cluster it chooses to join can influence the clusters’ utility. Therefore, we need to find a strategy for coalition formation and maintenance, i.e., the switch rule in Section 5, so as to optimize the social utility (defined as (13)), which is the sum of (8).

### 4.2. Main Properties of Coalition Formation Games

This involves a set of players, denoted by $\Omega =\left\{1,...,N\right\}$, who seek to form cooperative groups. Coalition formation games encompass coalition games where, unlike the canonical class, network structure and cost for cooperation play a major role. Therefore, we should determine to which coalition game the model belongs. The following demonstrates that our model is in good agreement with all of the requirements of the coalition formation game.

**Theorem** **1.** *The proposed coalition game does not satisfy the super-additive*.

**Proof.**  Super-additive implies that, given any two disjoint coalitions $S_{1}$ and $S_{2}$, if coalition $S_{1}\cup S_{2}$ forms, it can get more social utility than acting in $S_{1}$ and $S_{2}$ separately. Obviously, cooperation is always beneficial in a super-additive game. In other words, it will make the coalition structure have better social utility.

According to the coalition utility function in (8), the smaller utility is, the better the coalition formation is. Without loss of generality, we suppose that the players in $S_{1}$ are far from the players in $S_{2}$ for an actual scenario, which implies that the position function must be increased. Furthermore, the variations of the velocity function and the efficiency function are supposed to be zero. Therefore, we can know that:

(11) $$U\left(S_{1}\cup S_{2}\right)>U\left(S_{1}\right)+U\left(S_{2}\right)$$

Obviously, (11) demonstrates that such cooperation is not always beneficial. What is more, it is not easy to find a vehicle as the cluster head that could communicate with every vehicle in the coalition $S_{1}\cup S_{2}$. Hence, our proposed model does not satisfy the super-additive.  ☐

**Theorem** **2.** *The vehicles will seldom constitute a grand coalition*.

**Proof.**  According to the definition of efficiency function, the demanded bandwidth has to be close to the provided bandwidth by the head if we want to get lower efficiency function utility, which means that we expect more vehicles in a coalition. However, the number of vehicles is too large for the cluster head to satisfy their individual bandwidth requirement, because of the constrained resource of the head. Obviously, the coalition may have a meaningless existence when the following holds:

(12) $$B_{0}<\sum_{i\in hcluster}b_{i}$$

Subsequently, the vehicles in crossroads will make the positive decisions in our model to form an appropriate coalition structure instead of the grand coalition.  ☐

**Theorem** **3.** *The coalition structure is affected by the changeable scenario*.

**Proof.**  The coalition aggregation algorithm is iterative to obtain the final coalition structure with the best social utility. In this paper, the best utility means the lowest sum of all coalition utilities. Many factors in our model will affect the coalition utility, such as the joining or departure of any vehicles in any coalitions, as well as the changeable vehicle information (e.g., velocity, location and the demanded bandwidth). All of these factors should affect the overall coalition utility, which accordingly results in the variable coalition structure.  ☐

**Theorem** **4.** *The coalition structure is restricted by some external factors on the game*.

**Proof.**  According to the definition of coalition utility, some conditions must be satisfied during the formation game. The parameter *a* in (8) should be a constant, and the efficiency function of any coalition must be less than or equal to one. ☐

Therefore, we model the clustering strategy as a coalition formation game when the model proposed in this paper satisfies all of the above theorem.

### 4.3. Coalition Formation Concepts

The coalition formation game is used to find an optimal coalition structure when the grand coalition is no longer the optimal structure. By far, it has been applied to many scenarios, e.g., spectrum allocation in distributed cognitive radio networks in [35] and relay selection for cooperative cognitive radio networks in [34]. Inspired by the previous work, we formulate the problem proposed in this paper as a coalition formation game. At first, some important definitions about the coalition formation game are introduced.

**Definition** **4.** *A coalition partition is defined as the set* $\Pi =S_{1},...,S_{l}$, *which partitions the players’ set* Ω, *i.e.*, $\forall m,S_{m}\subseteq \Omega$ *are disjoint coalitions, such that* ${⋃}_{m=1}^{l}S_{m}=\Omega$.

**Definition** **5.** *We denote the social utility of any partition as the value of the coalition structure* Π, *which is the sum of all of the coalition utilities in the partition*.

(13) $$U\left(\Pi \right)=\sum_{m=1}^{l}U\left(S_{m}\right)$$

A number of partitions can be formed by *n* nodes in crossroads. The coalition formation game used here is to find the final partition with the best social utility. In our model, the best social utility must be the lowest one, because the coalition with lower utility represents a more stable and efficient cluster. Accordingly, it is necessary to analyze and compare the existing partitions to find the best coalition structure. Considering the social utility in (13), we now define the priority of coalition structures as below.

**Definition** **6.** *For any player i∈Ω, $S_{1}{≻}_{i}S_{2}$ implies that player i prefers being a member of coalition $S_{1}$$\left(i\in S_{1}\right)$ to being a member of $S_{2}$$\left(i\in S_{2}\right)$. We suppose the partition of $i\in S_{1}$ is $\Pi_{1}$ and the partition of $i\in S_{2}$ is $\Pi_{2}$. In this paper, the priority of any node i∈Ω is quantified as follows*.

(14) $$S_{1}{≻}_{i}S_{2}\Leftrightarrow U\left(\Pi_{1}\right)<U\left(\Pi_{2}\right)$$

According to Definition 6, a coalition structure is regarded as better than another if and only if the social utility could be decreased. The only difference between $\Pi_{1}$ and $\Pi_{2}$ is that the node *i* belongs to the different coalitions. Therefore, the priority also can be represented as below:

(15) $$\begin{array}{cc} S_{1}{≻}_{i}S_{2} & \Leftrightarrow U\left(S_{\Pi_{1}}\left(i\right)\right)+U\left(S_{\Pi_{2}}\left(i\right)\backslash \left\{i\right\}\right)\\ & <U\left(S_{\Pi_{2}}\left(i\right)\right)+U\left(S_{\Pi_{1}}\left(i\right)\backslash \left\{i\right\}\right) \end{array}$$

where $S_{\Pi_{n}}\left(i\right)$ represents the coalition that node *i* belongs to the partition $\Pi_{n}$. Clearly, any node could find an appropriate coalition to join via exploiting the proposed priority definition, and the coalition structure should tend to be more stable and efficient, as well. We will prove in the following section that the nodes in crossroads can achieve a stable coalition structure in a finite number of iterations by the coalition formation algorithm.

## 5. Coalition Formation Game-Based Clustering Strategy

In this section, we present the specific clustering strategy based on the coalition formation game in crossroads to create and maintain a stable and efficient cluster, including the cluster formation algorithm, cluster head election algorithm and clustering maintenance algorithm.

### 5.1. Coalition Formation-Based Clustering Strategy

We explain an algorithm based on the coalition formation game for forming clusters in this subsection. It should be clear that the most important for the coalition formation game is the strategy. Here, we adopt one basic rule, called the switch rule, to reach the final coalition structure with the most preferred order. The definition of the switch rule is below:

**Definition** **7.** *Switch rule: Given a partition $\Pi =S_{1},...,S_{l}$, a switch operation $S_{\Pi }\left(i\right)$ to $S_{m}\in \Pi$, $S_{m}\neq S_{\Pi }\left(i\right)$ is allowed for any vehicle i∈Ω, only if $S_{m}\cup i{≻}_{i}S_{\Pi }\left(i\right)$. In other words, the new partition $\Pi^{\prime }$ should have lower social utility than partition* Π, *which can be expressed as* $U\left(\Pi^{\prime }\right)<U\left(\Pi \right)$.

Accordingly, any vehicle can leave its current cluster $S_{\Pi }\left(i\right)$ and join another cluster $S_{m}\in \Pi$ if and only if $S_{m}\cup i$ is preferred compared to $S_{\Pi }\left(i\right)$. Besides, we suppose that the order that vehicles make switch decisions is random, and every vehicle has to know which clusters it can move in before starting the switch rule.

**Figure 2:**
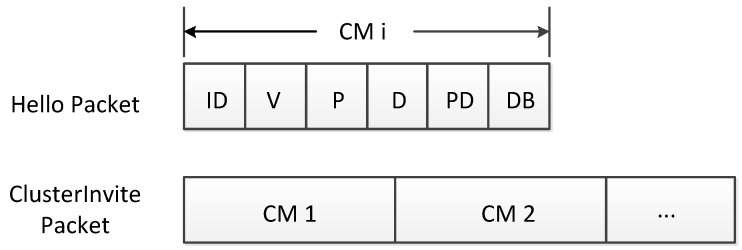
Packets’ content.

To achieve the goal, the nodes in crossroads have to exchange information among them. The CM should send its own information to the CHs over the **Hello** packet, while every CH will send **ClusterInvite** packet to its direct neighbors. Figure 2 explains the details of the **Hello** packet and the **ClusterInvite** packet. Concisely, the **Hello** packet contains its own ID, velocity, position, direction, provided bandwidth and demanded bandwidth, while the **ClusterInvite** packet contains all of the nodes’ information in its cluster. Based on this, node *i* may put the transmitters’ identification into its Direct Head List (DHL) when it receives **ClusterInvite** packets. Next, this node will employ (1) to calculate the angle between its direction and each transmitter’s direction. If $\theta >\frac{\pi }{4}$, the corresponding head is recognized moving in a different direction and deleted from its DHL. After checking *θ* and updating DHL, the remaining heads are the Direct Heads (DHs) that can communicate with node *i*.

In other words, the node *i* can move and join the clusters with the DHs. We Suppose that *h* is one of the DHs; $\Pi_{1}$ and $\Pi_{2}$ are the current partition with node *i* and the partition after switching to *h*, respectively. Clearly, *i* knows $S_{\Pi_{1}}\left(i\right)$ and $S_{\Pi_{1}}\left(i\right)\backslash \left\{i\right\}$, while *h* learns the corresponding utilities $U(S_{\Pi_{2}}\left(i\right))$ and $U(S_{\Pi_{2}}\left(i\right)\backslash \left\{i\right\})$, after implementing the head election mechanism introduced in the next part. In summary, node *i* will make a decision to switch to the *h* cluster.

The details of the clustering formation algorithm are shown in Algorithm 1. In the premise of the execution of Line 8, we will go back to Line 1 to continue searching the optimal clustering structure. When the network structure is stable (i.e., do not execute Line 8), the algorithm breaks the loop body and then updates the node status information in Line 12.

**Algorithm 1** Switch rule-based Clustering.**Require:** node i∈Ω; DHL set *φ*; cluster head h∈φ(i);
1:**for** i∈Ω **do**2:  Compute φ(i) ;3:  **for** j∈φ(i) **do**4:      Employ Algorithm 2 to select a cluster head;5:      Compute the related initial utilities via (8): $U_{i}=U\left(S_{\Pi_{1}}\left(i\right)\right)$, $U_{h}=U\left(S_{\Pi_{2}}\left(i\right)\backslash \left\{i\right\}\right)$;6:      Compute $U_{i2}=U\left(S_{\Pi_{1}}\left(i\right)\backslash \left\{i\right\}\right)$, $U_{h2}=U\left(S_{\Pi_{2}}\left(i\right)\right)$;7:      **if** $U_{i2}+U_{h2}<U_{i}+U_{h}$ **then**8:            *i* switches into cluster *h*;9:      **end** **if**10:  **end** **for**11:**end** **for**12:Update information after the network structure changes13:Go to Algorithm 4 to conduct cluster maintenance;


### 5.2. Cluster Head Election

With regard to (4), M(i) indicates the stability of node *i*, which can be compared with the threshold $M_{0}$ to identify whether the node can be considered as a cluster head. Although there may exist some nodes that satisfy the stable condition, in fact, we have to choose only one node from them to be treated as the CH. Consequently, we take into account the transmission capacity of them to choose the best node as the CH. The transmission capacity in this paper is defined by the bandwidth every node can provide. We assume that every vehicle is equipped with a hardware device that can be assigned bandwidth resources from the network. Among the alternative nodes, the node as the CH is selected with the largest available bandwidth.

When node *i* wants to switch to cluster *h* (*h* is the cluster head), it will calculate M(i) via (4). If $M\left(i\right)<M_{0}$, node *i* will turn to compare the transmission capacity with head *h*. If *i*’s available bandwidth is higher than *j*’s, *i* will be not only be merged with cluster *h*, but will also be set to the cluster head instead of *h* when executing the switch rule. After node *i* is set to the cluster head, $S_{\Pi }\left(i\right)$ will first choose a new CH in its current coalition for the purpose of implementation of the switch rule and intra- or inter-cluster data forwarding.

Algorithm 2 demonstrates the details of the cluster head election algorithm.

**Algorithm 2** Cluster head election.**Require:** node $i\in S_{1}$; cluster head $h\in S_{2}$; available bandwidth *p*;
1:**if** *i* wants to join in $S_{2}$ and p(i)>p(h) **then**2:  Calculate M(i) via (4);3:  **if** $M\left(i\right)<M_{0}$ **then**4:      *i* is set to the cluster head instead of *h*;5:  **end** **if**6:**end** **if**7:**if** *i* wants to leave $S_{1}$ and *i* is the head of current cluster **then**8:  Calculate $M\left(k\right),k\in S_{1}$;9:  $List=find(M\left(k\right)<M_{0}$);10:  Head=max(p(List));11:**end** **if**12:Update cluster head information;


The ideal situation is to suppose that none of the vehicles will lie about their transmission capacity to benefit from the model. Unfortunately, due to the ad hoc nature, malicious nodes can easily modify their provided bandwidth to escape or contend to be CHs and then corrupt networks and degrade social performance. Thus, one of the basic requirements for keeping the network operational is to reduce and isolate malicious nodes. This part will be introduced in the next section.

### 5.3. Clustering Maintenance

#### 5.3.1. Two Criteria for Finding Potential Heads

We suppose node *i* is a cluster member of head *h*. It has to reselect a cluster to join, once $\theta \left(i,h\right)>\frac{\pi }{4}$ or the distance of *i* and *h*, d(i,h), is beyond the predefined communication radius *D*. In Algorithm 1, node *i* needs to execute the switch rule with all of the heads in its DHL, which will result in a large amount of calculation. In order to reduce the expensive computational cost, we will introduce two criteria, the bandwidth criterion and the mobility criterion, limiting the heads in its DHL.

In general, different DHs represent different clusters. The bandwidth criterion is that we should ensure that the provided bandwidth is sufficient for the demand if node *i* switches into one cluster. Such a criterion is the postulate that node *i* should take into account before executing the switch rule. Accordingly, we can greatly reduce the amount of computation after detecting this criterion.

The mobility criterion is about the stay time of each node in one cluster. Considering the mobility of vehicles during driving, the stay time of a moving vehicle *i* with one of its DHs must be longer than that with its current head, so as to ensure that node *i* can join the most appropriate cluster. It is important to set up an ideal stay time of a cluster member to keep communicating with one of its DHs, so as to reduce the amount of computation during the iteration. Here, we will present the method to predict the ideal stay time of a cluster member.

Similar to [28], assume $v_{i}^{Ins}$, $v_{dh}^{Ins}$, $\left(x_{i}^{Ins},y_{i}^{Ins}\right)$ and $\left(x_{dh}^{Ins},y_{dh}^{Ins}\right)$ are the instantaneous velocity and position of the cluster member *i* and any one direct head DH, respectively. The instantaneous distance between *i* and DH can be represented as below.

(16) $$D^{Ins}=\sqrt{{\left(x_{i}^{Ins}-x_{dh}^{Ins}\right)}^{2}+{\left(y_{i}^{Ins}-y_{dh}^{Ins}\right)}^{2}}$$

Comparing the position and the velocity of the member *i* with those of the direct head DH, four different stay time prediction results, $T_{i,dh}^{stay}$, can be obtained:

(17) $$T_{i,dh}^{stay}=\left\{\begin{array}{cc} \frac{R+D^{Ins}}{v_{i}^{Ins}-v_{dh}^{Ins}} & \text{if }dh\text{ is in front of }i,v_{i}^{Ins}>v_{dh}^{Ins}\\ \frac{R+D^{Ins}}{v_{dh}^{Ins}-v_{i}^{Ins}} & \mathrm{if}\;dh\;\mathrm{is}\;\mathrm{behind}\;i,v_{i}^{Ins}<v_{dh}^{Ins}\\ \frac{R-D^{Ins}}{v_{i}^{Ins}-v_{dh}^{Ins}} & \mathrm{if}\;dh\;\mathrm{is}\;\mathrm{in}\;\mathrm{front}\;\mathrm{of}\;i,v_{i}^{Ins}<v_{dh}^{Ins}\\ \frac{R-D^{Ins}}{v_{dh}^{Ins}-v_{i}^{Ins}} & \mathrm{if}\;dh\;\mathrm{is}\;\mathrm{behind}\;i,v_{i}^{Ins}>v_{dh}^{Ins} \end{array}\right.,$$

where *R* is the communication radius of a mobile node. According to the mobility criterion, we can get a stable clustering structure for moving vehicles, and node *i* will execute the switch rule only if the direct heads can keep a longer communication duration. The details of finding the Potential Heads List (PHL) algorithm are shown in Algorithm 3.

**Algorithm 3** Find potential heads list.**Require:** node i∈Ω; i’s demand bandwidth D(i); DHL set φ(i); dh∈φ(i); the $cluster_{dh}$ demand bandwidth $D(cluster_{dh})$; dh’s provided bandwidth P(dh);
1:**for** dh∈φ(i) **do**2:  **if** $\left(D\left(i\right)+D\left(cluster_{dh}\right)\right)<P\left(dh\right)$||$\left(D\left(i\right)+D\left(cluster_{dh}\right)\right)<P\left(i\right)$ **then**3:      Calculate $T_{i,h}^{stay}$ and $T_{i,dh}^{stay}$;4:      **if** $T_{i,h}^{stay}<T_{i,dh}^{stay}$ **then**5:            $dh\in i^{\prime }s$ PHL;6:      **end** **if**7:  **end** **if**8:**end** **for**


**Figure 3:**
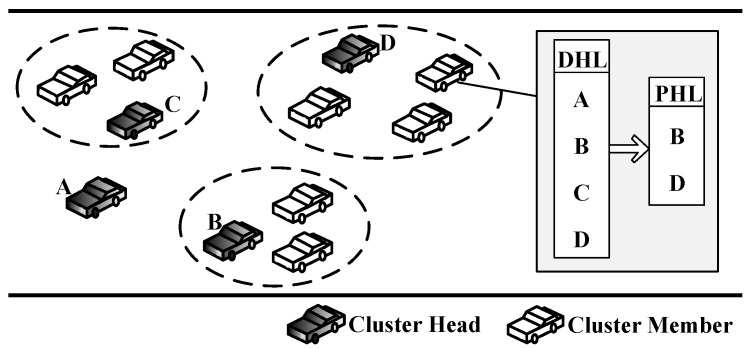
Example to save computational cost.

Next, we take Figure 3 as an example to illustrate how these criteria reduce the expensive computational cost.

In Figure 3, the DHL of the CM *f* consists of {A,B,C,D}, while its PHL contains {A,C}. Obviously, we learn that the two criteria surely reduce the computational cost. Thus, all of the members will certainly reduce the iteration number of the switch rule, which is described as (18).

(18) $$\prod_{i\in \Omega }\left(DH_{i}-P_{i}\right)$$

where $DH_{i}$ represents the number of heads in *i*’s DHL and $P_{i}$ means the number of heads in *i*’s PHL.

#### 5.3.2. Clustering Maintenance

We suppose node *i* is a cluster member of head *h*. It has to reselect a cluster to join, once $\theta \left(i,h\right)>\frac{\pi }{4}$ or the distance of *i* and *h*, d(i,h), is beyond the predefined communication radius *D*. In addition, we also propose the reputation-based incentive and penalty mechanism in the following section, which also runs during the maintenance process, to solve the selfish nodes in the system. In other words, the cluster maintenance scheme is illustrated as Algorithm 4.

**Algorithm 4** Cluster maintenance.**Require:** node $i\in S_{1}$; cluster head $h\in S_{1}$; there is *N* nodes in Ω;
1:Start reputation based on incentive and penalty mechanism during data transmission2:**for** i∈Ω **do**3:  **if** $\theta \left(i,h\right)>\frac{\pi }{4}$ or d(i,h)>D **then**4:      $S_{1}=S_{1}\backslash \left\{i\right\}$;5:      *i* becomes isolated nodes;6:      Employ Algorithm 3 to obtain node *i*’s PHL set δ(i);7:      **for** h∈δ(i) **do**8:            Recompute $U_{i}$ and $U_{h}$;9:            Employ Algorithm 2 to select a cluster head;10:            Recompute $U_{i2}$ and $U_{h2}$;11:            **if** $U_{i2}+U_{h2}<U_{i}+U_{h}$ **then**12:                    *i* switches into cluster *h*;13:            **end** **if**14:      **end** **for**15:  **end** **if**16:**end** **for**
17:Update nodes’ information;


### 5.4. Convergent Proof for the Proposed Algorithm

**Theorem** **5.** *Starting from any given initial coalitional partition, nodes will all converge to a final partition $\Pi_{f}inal$ composed of a number of disjoint coalitions through the proposed algorithm*.

**Proof.**  Through the analysis of the switch rule in Algorithm 1, we can find that the switch operation will lead to either an unvisited partition or a visited partition. When node *i* conducts the switch rule, it will choose whether to join a new coalition or stay in the current coalition. A new partition will be created based on the switch operation only if node *i* decides to join the coalition. Due to the finite set of players, the number of partitions is also limited. Thus, the sequence of switch operations will always end at a final partition $\Pi_{f}inal$ after detecting all of the sets, which completes the proof.  ☐

**Definition** **8.** *A partition* $\Pi ==S_{1},...,S_{l}$ *is Nash-stable if* $\forall i\in \Omega ,S_{\Pi }\left(i\right){≻}_{i}S_{m}\cup \left\{i\right\}$ *for all* $S_{m}\in \Pi$

**Theorem** **6.** *Any partition $\Pi_{f}inal$ resulting from the proposed algorithm is Nash-stable*.

**Proof.**  If the final partition $\Pi_{f}inal$ is not Nash-stable, there must exist a node i∈Ω and a coalition $S_{m}\in \Pi_{f}inal$, such that $S_{m}\cup \left\{i\right\}{≻}_{i}S_{\Pi_{final}}\left(i\right)$. Consequently, node ican perform a switch operation, which contradicts the fact that $\Pi_{f}inal$ is the final partition. Therefore, we complete the proof.  ☐

## 6. Reputation-Based Incentive and Penalty Mechanism

The previous studies in the field of network security, such as [36,37,38], revealed that the inside attacks by the authorized nodes are far more difficult to control than the outside attacks by the unauthorized nodes. Let us recall that our proposition is to form an efficient and stable clusters with the nodes in crossroads. Nevertheless, as mentioned in Section 3, some selfish vehicles may tell a lie about the bandwidth they can provide so as to benefit from the model [39], which will seriously degrade the efficiency of clusters. Therefore, it is important to create and maintain clusters carefully. Our basic motivation is to mitigate the selfishness problem based on the proposed incentive and penalty mechanism.

### 6.1. Basic Definition and Assumption

In the previous section, we have presented a stable and efficient switch rule-based clustering and maintenance algorithm. However, the cluster security becomes a key issue for VCPS [40,41,42], especially when nodes in clusters become malicious with the intention to get more bandwidth. Such a black deed will affect the spread of the traffic information on the road or occupy the bandwidth allocated to other users for entertainment. Definition 9 provides the definition of malicious nodes in this paper.

**Definition** **9.** *Malicious nodes: the nodes that falsely declare their transmission capacity to make a profit*.

Thus, a way to guarantee security and strong clusters is needed. The proposed mechanism should adhere to the following assumptions.

1. We assume that the head will allocate bandwidth for itself firstly and then for the cluster members. Each node needs to pay for the bandwidth allocated for itself. Furthermore, the charge for the bandwidth is calculated by the amount of access and the node reputation in the cycle $T_{s}$.
2. Reputation values, affecting the cost level of nodes in the system, are defined as −1, 0 and +1, which represent the high, normal and low cost level, respectively.
3. The node should be blacklisted from the model if its reputation is −1 for two consecutive cycles.

Accordingly, we will describe the cumulative cost of node *i* as $T_{s}*b_{i}*\left(level_{1}+level_{2}+level_{3}+...\right)$ when using $b_{i}$ to be the bandwidth demand of the node. Here, $level_{n}$ represents the cost standard in the *n*-th cycle. The target of each node is to minimize the cost for bandwidth on the basis of satisfying its own requirement.

### 6.2. Incentive and Penalty Mechanism

#### 6.2.1. Cluster Head

Although there are some conveniences as CHs, in general, every node in the network is unwilling to serve as a head due to the heavy tasks on communication and management. To be honest, some selfish nodes may report less bandwidth than they can provide in their Hellopackets, so as to escape from serving other nodes. In that case, we intend to design an incentive scheme by rewarding CHs, to encourage nodes to tell the true bandwidth they have. In particular, we will define the initial reputation of the CHs as +1, while those of the CMs are zero.

Such an incentive is so attractive that all of the nodes want to become the CH because of the high reputation. However, we have to pay more attention to the malicious nodes that provide a false bandwidth in Hello packets to win the benefits. That black deed should result in the worst communication performance either within a cluster or among clusters. Accordingly, we introduce some penalties into the clustering strategy to prevent the selfishness. Specifically, those CMs that cannot obtain enough bandwidth (also called bandwidth crisis) will send an Accusationpacket to the RSUs (a reliable reputation center) for the purpose of reporting the selfishness of the CH. In the strategy, the reputation of the CH will be turned to −1 if there exists such an accusation.

#### 6.2.2. Cluster Member

The CMs will keep their cost at the normal level when they successfully accuse their CH. Though we can monitor the malicious behavior of the cluster head via the above method, the selfishness of cluster members can also induce them to tell a lie about the nonexistent bandwidth crisis, so as to increase their possibility of being CHs. In order to solve such a selfishness attack resulting from CMs, we let the RSUs monitor the CHs that are being accused. Specifically, the adjacent RSU will examine the real bandwidth that the CH provides. If the RSU considers that the CH cannot provide the bandwidth it previously told, its reputation will be turned to −1. Otherwise, the reputation of the CM that sends the Accusation packets will be set to −1 because of the false reporting bandwidth crisis.

**Figure 4:**
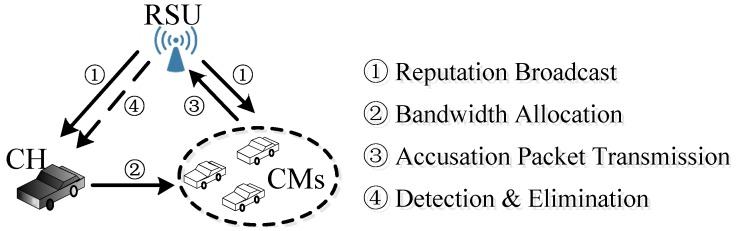
The reputation-based incentive and penalty mechanism.

The four steps of the protocol are shown in Figure 4. First, the RSU broadcasts the initial reputation to both CH and CMs. Then, the CH allocates bandwidth based on the demand of every CM. Next, the CMs make a decision on sending an accusation packet or not. Finally, the RSU monitors the CH and broadcasts the practical reputation for all nodes.

Adopting the reputation-based incentive and penalty mechanism in the cluster maintenance process can perfectly keep the clusters from the selfishness problem. Intuitively, the nodes with low reputation for two consecutive cycles will be removed from the current cluster and cannot join the process of clustering maintenance. Therefore, all nodes have to tell the truth to obtain higher reputation, as well as a lower cost level.

## 7. Performance Evaluation

In this section, we will firstly evaluate the performance of the proposed clustering strategy from the perspectives of stability, efficiency and computation cost. The existing schemes, such as MOBIC [23], EnLOSC [28] and our previous scheme CG-bCS [33], are implemented as a comparison to analyze the performance of our strategy, CG-SECC. Besides, we also give a case study to illustrate the security of the reputation-based incentive and penalty mechanism.

### 7.1. Numerical Evaluation

The simulation scenario is a two-lane crossroad, as shown in Figure 1. A variety of packets shown in Figure 2, containing self information of every node in VCPS, can be broadcast, forwarded and shared among vehicles. For more practical considerations, the number of vehicles is set up from 20 to 250, and the vehicle speed range is selected randomly between 0 km/h and 36 km/h; the provided available bandwidth and the demand bandwidth are defined as 10∼50 Mbit/s and 1∼10 Mbit/s, respectively. Besides, the communication radius *D* is 100 m, and the threshold $M_{0}$ to identify whether a node can be considered as a cluster head is 10.

Unlike the fast-changing highway scenario, we can adopt the classification method instead of machine learning to reduce the computation delay for obtaining appropriate $k_{1}$, $k_{2}$ and *a* due to the stable traffic flow in our system model (i.e., the steady and low-speed moving in the crossroad [43]). In that case, we can ignore the delay caused by the the parameters selection in (8). Here, the initial $k_{1}$ and $k_{2}$ are setup as one and 0.1, while the initial *a* is denoted as 1.5, to conduct a similar simulation scenario as in [33] in terms of the velocity and the vehicles’ position. When the clustering performance degrades dramatically, the coefficients values of the utility must be adjusted based on the classification method in Section 4 to satisfy the changeable environment.

**Figure 5:**
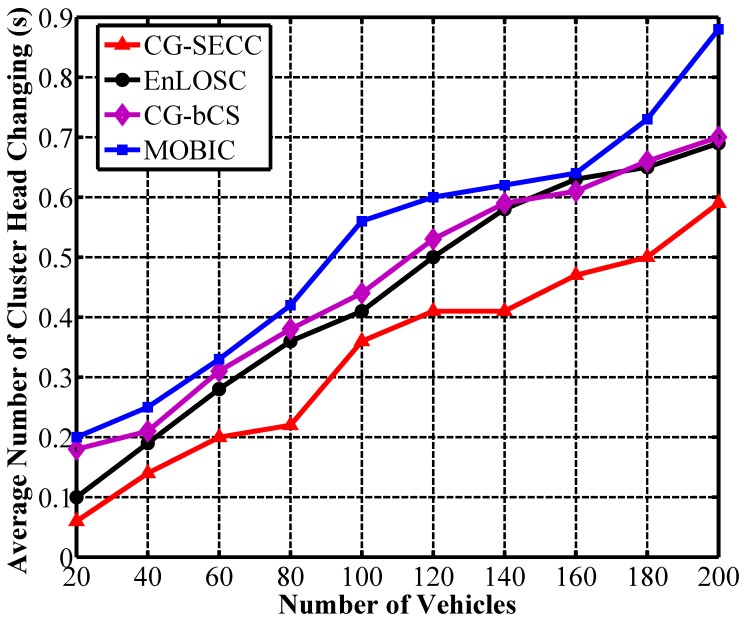
Analysis of cluster stability.

Figure 5 shows the performance of clustering stability represented by the average number of cluster heads changing per second. Obviously, the cluster-based communication network will not be stable if the value is large. Among the four algorithms, CG-bCS, EnLOSC and our proposed strategy can obtain more stable clusters than the MOBIC scheme due to the election of proper clustering heads. Besides, in comparison with CG-bCS and EnLOSC from Figure 5, the number of CH handoffs in CG-SECC is fewer, which represents higher clustering stability. The reason for the improvement of clustering stability is the application of the nodes’ mobility criterion. Moreover, the better stability also indicates that the proposed strategy in this paper can solve the frequently changeable network topology challenges causing by the nodes’ high mobility. In other words, the proposed strategy demonstrates the high stability so as to be more suitable for the crossroad scenario in VCPS.

**Figure 6:**
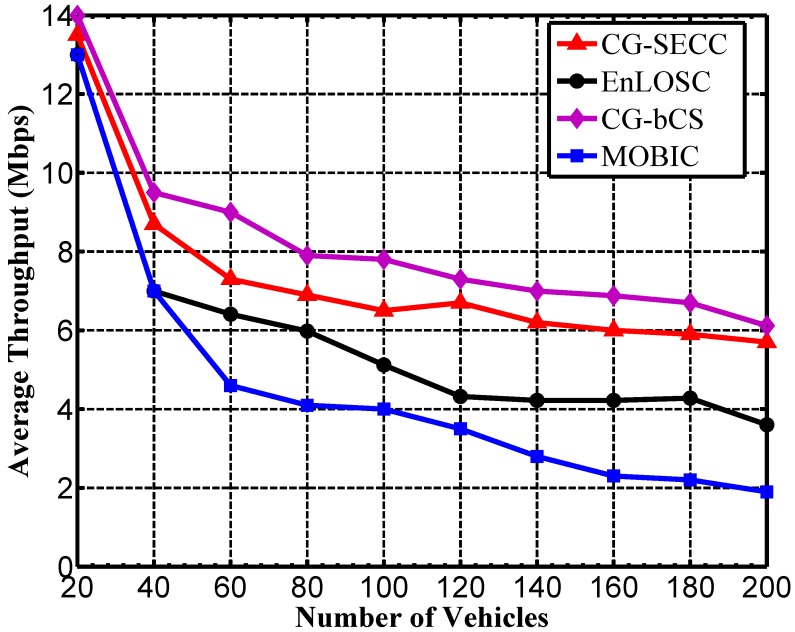
Analysis of the throughput.

For the purpose of illustrating the efficiency of clusters, we explore average throughput per vehicle in Figure 6. Obviously, the cluster is efficient if this average value is large. The average throughput of every vehicle is merely equal when the vehicle density is low, because the provided available bandwidth from cluster heads is enough for the requirements of all members. Once the density is high, however, the overload issue for cluster heads may frequently occur. Depicted in Figure 6, apparently, the average throughput is the lowest in MOBIC. The result represents that we may lose efficiency due to the overload of the cluster head if we consider only the stability of the cluster. In other words, the algorithm considering the overhead of the cluster head is especially necessary. In addition, we can also learn that the cluster efficiency of both CG-bCS and the proposed strategy is higher than that of EnLOSC, which illustrates that clustering strategies can precisely decrease the cluster heads’ overhead through the bandwidth on demand method. Moreover, comparing with CG-bCS, CG-SECC has a slightly lower clustering efficiency. This is due to the cost of the clustering stability and the reduced calculation. In other words, when we use the two criteria in Section 5.3 for finding potential heads to decrease the calculation for nodes, we may not obtain the optimal clustering structure, which finally results in the slightly lower efficiency of clusters.

**Figure 7:**
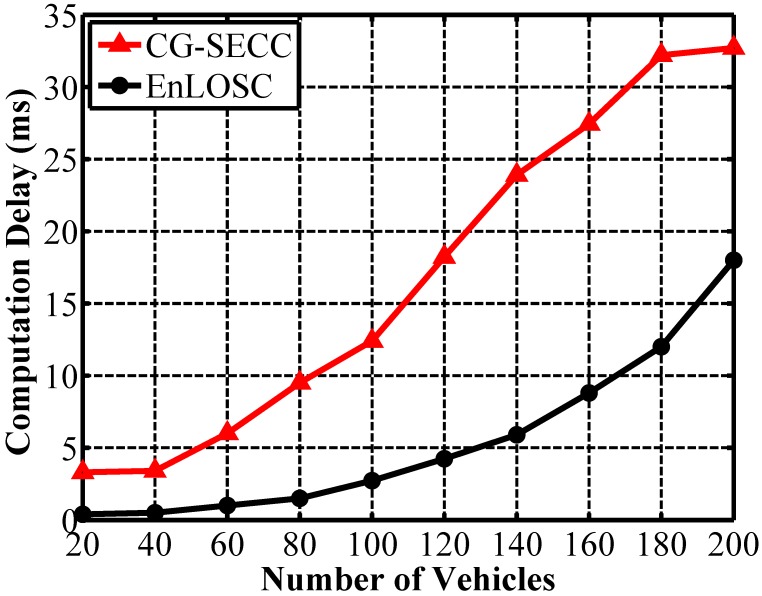
Analysis of calculated delay for clustering formation.

Due to the frequently changing topology structure of VCPS, the computation complexity for clustering formation should be adaptive to reduce the end-to-end delay. Figure 7 provides the computation delay for clustering formation and maintenance using CG-SECC and EnLOSC. Note that we omit the computational analysis of MOBIC because of its weak performance in the aspect of cluster stability and the average throughput. In Figure 7, we learn that the computational delay in CG-SECC is adaptive, but larger than that in EnLOSC, which is clearer along with the increasing number of vehicles. However, this increased delay is the cost of the improvement of stability and efficiency for the clustering structure. In other words, in order to prolong the lifetime of a cluster, we surely need a more stable and efficient clustering structure.

Moreover, we also demonstrate the computational complexity between CG-bCS and the proposed strategy. Table 1 shows the analysis of the iteration times when the number of vehicles, *N*, increases. Obviously, the number of iterations for these two algorithm is small when there are only a few vehicles in the crossroads (i.e., *N* is small), because of the limited cooperation possibility. Furthermore, we can conclude that the number of iterations that CG-SECC needs to converge is much smaller than that of CG-bCS. This is owing to the use of two criteria for finding potential heads, which sharply decreases the cluster heads with which nodes want to cooperate.

Actually, the main complexity of the clustering maintenance scheme is caused by both the amount of computation in one iteration and the number of iterations. To compare the complexity between CG-bCS and the scheme in this paper, we consider the following two aspects. On the one hand, we suppose the common pseudocode from Line 8 to Line 13 in Algorithm 4 for electing the cluster head and utility calculation as a unit of calculation, called the Inner Loop Part (ILP). Accordingly, in each iteration of CG-bCS, the complexity of cluster maintenance can be represented as $\sum_{i=1}^{N}DHL\left(i\right)*\mathrm{ILP}$. Similarly in CG-SECC, the main complexity of the clustering maintenance is represented as $\sum_{i=1}^{N}PHL\left(i\right)*\mathrm{ILP}$. Obviously, the number of nodes in $i^{\prime }s$ DHL is definitely more than $i^{\prime }s$ PHL. Thus, the complexity of the strategy in this paper is lower than CG-bCS. On the other hand, due to the lesser number of iterations of CG-SECC shown in Table 1, the amount of computation in CG-SECC is reduced greatly. All in all, although the proposed strategy may not create the coalition structure with optimal efficiency, it can implement fast clustering with the average throughput similar to CG-bCS.

Through these three numerical evaluations and analysis, we can conclude that the proposed strategy with the lower number of iterations is more adaptive to obtain the clustering structure in the crossroads.

### 7.2. Case Study

The security problem in VCPS has already been studied in the past. In [42], a Bayesian coalition game-based reliable data transmission was proposed for the vehicular cloud, which focused on the packet delivery ratio, end-to-end delay and reliable data transmission. Then, they also exploited a new approach, named VDDZ, to protect the CA in the cluster from direct communications and attacks from unknown vehicles in [40]. Although these studies could ensure the data consistency and legitimacy enforcement of messages, both of them cannot keep the malicious nodes from falsely declaring their transmission capacity. In fact, the reputation-based incentive and penalty mechanism proposed in Section 6 is not only aware of and deals with such malicious nodes, but also enhances the effectiveness of the clustering structure. To evaluate the proposed incentive and penalty mechanism, this section exploits a case study to analyze the related secure performance.

**Figure 8:**
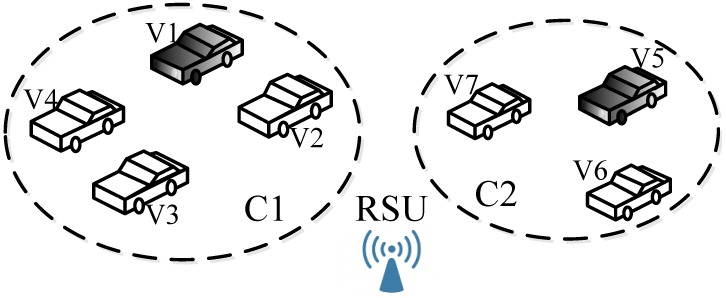
Considered clustering case.

Figure 8 provides two existing clusters (C1 and C2) with the determined CHs (V1 and V5). Here, we suppose that the provided bandwidth of V1 is 12 Mbps, and the demands of the nodes in C1 (i.e., V1, V2, V3 and V4) are 1, 2, 3 and 4 Mbps. Such a bandwidth will be used for knowing the road traffic condition, accident prevention, payment services or entertainment applications. Furthermore, V7 in C2 is assumed as a malicious one. In other words, V7 can only provide an 8-Mbps bandwidth, but it will report the fake bandwidth capability for the purpose of becoming the CH and obtaining better reputation.

We first analyze the security in the existing cluster (e.g., C1). Supposing that V2 acts as a malicious node, it will send an accusation packet to the RSU to replace V1 as the new cluster head. Obviously, such selfish behavior will heavily decrease the current cluster’s efficiency. Thus, we can exploit the reputation-based incentive and penalty mechanism to effectively prevent this from happening. In that case, the final reputation table of all of these nodes is depicted in Table 2.

Next, once the PHL of the malicious vehicle (V7) includes V1, it will broadcast its fraud bandwidth capability (e.g., 13 Mbps) in its Hello packet to contend to be selected as a cluster head so as to obtain higher reputation. As mentioned previously in Section 5, V7 will have the opportunity to replace V1 as the next cluster head. Obviously, the bandwidth V7 provides must not satisfy the total demand of the nodes in C1, which will cause the degradation of the clustering communication. Particularly, some CMs (e.g., V2) in the cluster cannot complete their information transmission. Thus, we start the incentive and penalty mechanism. Namely, V2 will send an Accusation packet to the adjacent RSU to report the fraud capability of V7. After detecting the real provided bandwidth of V7 by the adjacent RSU, the final reputations of every node in C1 are listed in Table 3.

Without using the reputation-based incentive and penalty mechanism, in fact, the real average throughput of every vehicle in C1 is only 1.6 Mbps, while that value after adopting the mechanism will be up to 2.4 Mbps. Note that different cases can be handled in the same way.

In other words, this case study aims at verifying whether our mechanism could (1) detect and isolates malicious nodes and (2) improve the clusters performance. According to the above analysis, we learn that the reputation of malicious nodes is set as −1 and that the value of cluster heads is rewarded as +1. Besides, we can conclude that the reputation-based incentive and penalty mechanism will improve the average throughput of the cluster.

## 8. Conclusions

In this paper, we have proposed a novel clustering algorithm for VCPS based on the coalition formation game to address the overload challenge for the cluster head in a crossroad scenario. To obtain the optimal clustering solution of the tradeoff between the stability and efficiency of clusters, the overall coalition utility is defined as the sum of three factors, including the velocity function, the position function and the efficiency function. The vehicles, denoted as the nodes in the model, could make a decision about whether to switch to a new coalition or to stay in the current coalition. By theorem proving, the coalition formation strategy can converge to a final Nash-stable partition. Moreover, we introduce two criteria into the clustering strategy to reduce the expensive computational cost and propose the reputation-based incentive and penalty mechanism to report and record malicious nodes so as to ensure system reliability, as well as enhance the clusters’ average throughput. Numerical simulation results and a case study demonstrate that the proposed algorithm not only improves the clustering stability and efficiency, but also has the capability to reject the malicious nodes. In the future, we intend to study the information sharing security issues in the VCPS and will introduce the related security strategy to the clustering strategy.

## Figures and Tables

**Table 1:** Number of iterations.

Vehicle Number	20	60	100	140	180	220
CG-bCS	80	720	3540	8403	14505	20729
CG-SECC	14	101	310	701	1229	2043
The Saved Proportion	82.50%	85.97%	91.24%	91.66%	91.53%	90.14%

**Table 2:** Reputation for malicious accusation.

Vehicles	1	2	3	4
Reputation	+1	−1	0	0

**Table 3:** Reputation for fraud capability.

Vehicles	1	2	3	4	7
Reputation	+1	0	0	0	−1
